# Game-Based Assessment of Peripheral Neuropathy Combining Sensor-Equipped Insoles, Video Games, and AI: Proof-of-Concept Study

**DOI:** 10.2196/52323

**Published:** 2024-10-01

**Authors:** Antao Ming, Vera Clemens, Elisabeth Lorek, Janina Wall, Ahmad Alhajjar, Imke Galazky, Anne-Katrin Baum, Yang Li, Meng Li, Sebastian Stober, Nils David Mertens, Peter Rene Mertens

**Affiliations:** 1 University Clinic for Nephrology and Hypertension, Diabetology and Endocrinology Otto von Guericke University Magdeburg Magdeburg Germany; 2 University Clinic for Neurology Otto von Guericke University Magdeburg Magdeburg Germany; 3 Pure-systems Magdeburg Germany; 4 Affective Neuroscience Lab Friedrich Schiller University Jena Jena Germany; 5 Artificial Intelligence Lab Otto von Guericke University Magdeburg Magdeburg Germany

**Keywords:** diabetes mellitus, metabolic syndrome, peripheral neuropathy, sensor-equipped insoles, video games, machine learning, feature extraction

## Abstract

**Background:**

Detecting peripheral neuropathy (PNP) is crucial in preventing complications such as foot ulceration. Clinical examinations for PNP are infrequently provided to patients at high risk due to restrictions on facilities, care providers, or time. A gamified health assessment approach combining wearable sensors holds the potential to address these challenges and provide individuals with instantaneous feedback on their health status.

**Objective:**

We aimed to develop and evaluate an application that assesses PNP through video games controlled by pressure sensor–equipped insoles.

**Methods:**

In the proof-of-concept exploratory cohort study, a complete game-based framework that allowed the study participant to play 4 video games solely by modulating plantar pressure values was established in an outpatient clinic setting. Foot plantar pressures were measured by the sensor-equipped insole and transferred via Bluetooth to an Android tablet for game control in real time. Game results and sensor data were delivered to the study server for visualization and analysis. Each session lasted about 15 minutes. In total, 299 patients with diabetes mellitus and 30 with metabolic syndrome were tested using the game application. Patients’ game performance was initially assessed by hypothesis-driven key capabilities that consisted of reaction time, sensation, skillfulness, balance, endurance, and muscle strength. Subsequently, specific game features were extracted from gaming data sets and compared with nerve conduction study findings, neuropathy symptoms, or disability scores. Multiple machine learning algorithms were applied to 70% (n=122) of acquired data to train predictive models for PNP, while the remaining data were held out for final model evaluation.

**Results:**

Overall, clinically evident PNP was present in 247 of 329 (75.1%) participants, with 88 (26.7%) individuals showing asymmetric nerve deficits. In a subcohort (n=37) undergoing nerve conduction study as the gold standard, sensory and motor nerve conduction velocities and nerve amplitudes in lower extremities significantly correlated with 79 game features (|*R*|>0.4, highest *R* value +0.65; *P*<.001; adjusted *R*^2^=0.36). Within another subcohort (n=173) with normal cognition and matched covariates (age, sex, BMI, etc), hypothesis-driven key capabilities and specific game features were significantly correlated with the presence of PNP. Predictive models using selected game features achieved 76.1% (left) and 81.7% (right foot) accuracy for PNP detection. Multiclass models yielded an area under the receiver operating characteristic curve of 0.76 (left foot) and 0.72 (right foot) for assessing nerve damage patterns (small, large, or mixed nerve fiber damage).

**Conclusions:**

The game-based application presents a promising avenue for PNP screening and classification. Evaluation in expanded cohorts may iteratively optimize artificial intelligence model efficacy. The integration of engaging motivational elements and automated data interpretation will support acceptance as a telemedical application.

## Introduction

For primary care clinicians, it is a challenge to diagnose peripheral neuropathy (PNP) due to its diverse forms and presentations. Especially, in patients with diabetes mellitus or metabolic syndrome (MetS), the onset is insidious, and most affected individuals are not aware of distal, often symmetric PNP [1,2]. Further common causes consist of excessive and chronic alcohol use, nerve injury, toxin exposure, nutritional deficiencies, chemotherapy, and genetic disorders [3].

According to the International Diabetes Federation, more than 435 million people worldwide are diagnosed with diabetes, which is expected to rise to 693 million in 2045 [4]. A total of 25% to 50% of patients with diabetes are impacted by PNP, and the severity varies according to age, duration of diabetes, and level of glucose control [3,5,6]. PNP significantly impacts the mobility of patients and is associated with disturbed gait and coordination, increased likelihood of falls, higher incidence rates of diabetic foot syndrome, and frail mental health [1]. Peripheral nerves encompass large nerve fibers (Aα- and Aβ-fibers; vibratory and pressure sensation) and small nerve fibers (unmyelinated C- and Aδ-fibers; pain and warm or cold sensation) [7,8]. Besides diminished sensation, PNP may also cause involuntary symptoms of discomfort and pain. Complications such as tissue damage, infections, and ultimately minor and major foot ulcerations are estimated to affect 19% to 34% of individuals with diabetes during their lifetime, especially with delayed diagnosis of PNP or inadequate preventive measures [9]. Notably, every fifth moderate to severe diabetes-related infection will prompt lower extremity amputation [9,10]. On the other hand, 4 of 5 amputations may be prevented by adequate podiatry care [11].

Due to their invasive nature, nerve conduction studies (NCSs) and skin biopsies are not applicable as screening tools [12]. According to the position statement published already 15 years ago by the American Diabetes Association, “assessment should include a careful history and either temperature or pinprick sensation [...] and vibration sensation [...]” [5,11]. National Institute for Health and Care Excellence [13] recommends vibration perception testing using a 128-Hz tuning fork together with a 10-g (Semmes-Weinstein) monofilament for the screening of PNP. However, neuropathy testing is infrequently provided to patients at high risk, possibly due to potential barriers such as the unavailability of health care (restrictions on facilities, care providers, or time), patient-related factors (absence of pain sensation in feet, lack of understanding of diabetes, and its possible complication), and differences in health care system [14]. The implementation of clinical testing for PNP by monofilament and vibration sensation tests is still not optimal. First, numbers of testing are as low as 35% in high-risk cohorts [15]. Second, these tests identify diabetic peripheral neuropathy at a late, irreversible stage [16,17]. An abnormal monofilament test is associated with a high 3-year relative risk of foot ulceration or lower limb amputation, reaching 15% (95% CI 9.0-26.0) [18]. Although small fibers undergo early and progressive injury in diabetes, their assessment is not included in annual diabetic foot screening programs [19,20]. Some innovations as point-of-care devices have been brought forward; however, they are not established as routines, such as DPN Check (large fibers), NeuroQuick, NeuroPad, Corneal Confocal Microscopy, and Sudoscan (small fibers) [1].

All these aspects urge for the optimization of PNP diagnosis, possibly even prevention and management. Up to 50% of affected individuals remain asymptomatic, while the remainder develop numbness, tingling, pain, or weakness [5,21]. Symptoms commonly originate distally (ie, at the tips of the toes) and spread proximally with a symmetric distribution. Unilateral or asymmetric forms of PNP (eg, diabetic radiculoplexus neuropathy) have also been reported [22]. Further investigation of PNP screening techniques needs to address these challenges.

Recently, using gamification in health assessments combined with wearable sensors has garnered great attention [23,24]. The integration of game-like elements can enhance user engagement, leading to heightened motivation and increased adherence to health goals. Moreover, the continuous data acquisition capabilities of wearables facilitate real-time health monitoring, providing users with instantaneous feedback and enabling more precise trend analysis [25,26]. This synergistic approach holds the potential to instigate positive behavioral changes, ensuring that individuals are not only informed about their health status but are also incentivized to maintain or improve it. Therefore, our proof-of-concept study was designed to identify patients at risk for PNP in a playful approach and further enable timely intervention to prevent complications [27].

The primary objectives of this study were to develop and evaluate a playful assessment device for peripheral nerve function through video-based games that are coordinated through the feet with pressure sensor–equipped insoles.

Specifically, we (1) designed a system with real-time transfer of pressure recordings to a tablet application for gaming control and a central database for analysis. (2) Video-based games were designed with varying difficulties. (3) Feature extraction methodologies were set up to explore data analysis from a pilot study. Herein, the game-based data set was correlated with NCS and clinical findings. Two ways were followed: predefined hypothesis-driven parameter analyses such as reaction time, sensation, skillfulness, endurance, balance, and muscle strength as well as feature extraction. (4) Optimization of the data analyses was performed by training and validation data sets to yield classification models.

## Methods

### Ethical Considerations

The study was conducted following approval of the study protocol by the local ethical committee of the Otto von Guericke University Magdeburg (approval 28/17; March 13, 2017). All study participants provided written informed consent upon a detailed explanation of the study protocol, test procedure, and data policy. No compensation was provided for participants except for travel costs. Information regarding study participant identities was removed. The pseudonymized study data were used for analyses. The figures provided in this work are deidentified.

### Study Design and Recruitment Procedures

In this proof-of-concept exploratory cohort study, the game platform development and clinical study were carried out at the University Clinic for Nephrology and Hypertension, Diabetes and Endocrinology at the Otto von Guericke University Magdeburg, Germany.

Patients were recruited from July 2020 to January 2023 from the university outpatient clinic, which is specialized in care for comorbidities in patients with diabetes and MetS. A questionnaire about past medical history, diabetes mellitus history (duration, type, treatment, sensory disturbances, complaints, and movement restrictions in daily life), autonomic diabetic neuropathy (dizziness, heart rate arrhythmia, urination disorders, and sweating function), diabetes-associated comorbidities, and daily activities (sports, handedness, and dominant foot) was performed. A physical examination with bedside neurological and sensory nerve testing was performed by a study physician with a standardized evaluation to identify symptoms and signs. The Montreal Cognitive Assessment (MoCA) or Mini Mental State Examination (MMSE) was performed to determine and quantify the cognitive capabilities of the participants.

Patients with diabetes mellitus or MetS aged between 18 and 85 years were eligible for the study, except for those with the following conditions: macroangiopathy of the lower extremities (ankle-brachial index <0.9 or ≥1.3 in either leg), physical deformities (amputations and foot and leg deformities requiring orthopedic shoe fitting), manifest neuropathic foot ulceration, visual disorders including visual acuity of less than 0.8 (except for corrected myopia and hyperopia), muscular diseases or motor diseases, myocardial infarction <12 weeks, heart failure (New York Heart Association III or IV), transient ischemic attack, stroke, tremor, lack of ability to give consent for any reason, and lack of ability to use a mobile phone. Patients with painful diabetic neuropathy, clinical severe autonomic neuropathy (eg, chronic diarrhea), and other chronic pain conditions (necessitating painkillers) that may impair the ability to perform study-related tasks or vestibular disorders (with signs of vertigo) were not evaluated in the study. We opted to exclude these patients, as our study was intended as a proof-of-concept study to generate a basis for predictive scores of PNP.

A study physician meticulously detailed the study protocol, test procedures, and data policy to the patients deemed eligible. Subsequently, these patients were enrolled in the study upon the provision of written informed consent. Compensation was not paid to the study participants except for travel costs.

### PNP Assessment

PNP screening was performed by a study physician with a standardized evaluation to identify symptoms and signs. The neuropathy symptom score (NSS) was used to summarize the type, localization, severity, and exacerbation of neuropathic symptoms on a scale ranging from 0 to 10, with more points indicating more severe symptoms (categorization: 3-4=mild, 5-6=moderate, and 7-10=severe symptoms) [28]. The neuropathic signs were classified with the most widely used and accepted neuropathy disability score (NDS, 0-10 points) that included examination of vibratory perception (using a 128-Hz tuning fork, measurement dorsal on big toe joint, 0=normal and 1=reduced or absent), temperature sensation (using a Tip Therm; Tip Therm GmbH, measurement on the dorsum of the foot, 0=normal and 1=reduced or absent), pain sensation (using pinprick, measurement on the dorsum of the foot, 0=normal and 1=reduced or absent), and ankle reflex (0=normal and 1=reduced or absent) [29]. According to the NDS score, the severity of neuropathy was graded as follows: 3-5=mild, 6-8=moderate, and 9-10=severe. In addition, a monofilament test was performed on 3 test sites on the dorsum of the feet (using 10-g Semmes-Weinstein monofilaments, 0=no insensate sites and 1=with ≥1 insensate sites) [30].

According to the aforementioned physical examinations, we defined PNP as 2 or more positive findings related to neuropathic symptoms or signs (vibration perception, temperature sensation, pain sensation, ankle reflex, and pressure sensation evaluated by the monofilament testing) per foot side [31]. Our definition of PNP is equal to “probable” neuropathy introduced in the Toronto Consensus Guidelines [32]. Based on the assumption that asymmetric (unilateral) PNP may be observed in patients with diabetes [22] and the impact on game outcomes is uncertain, we analyzed data from left and right feet separately. Asymmetric PNP was defined as at least 1 asymmetric finding in the clinical examination. Small fiber neuropathy (SFN) was diagnosed when pain sensation (pinprick) or temperature sensation was reduced or absent [7]. Large fiber neuropathy (LFN) was diagnosed with abnormal results of vibration perception, monofilament testing, or Achilles tendon reflexes [8].

For a subcohort of participants, neurophysiological testing using NCS [33] was carried out by board-certified neurologists in the University Clinic for Neurology, Otto von Guericke University Magdeburg. Nerve conduction velocities (NCVs) and amplitudes were quantified for sensory (sural) and motor (tibial) nerves in the lower extremities.

### Study Cohorts

**Figure 1:**
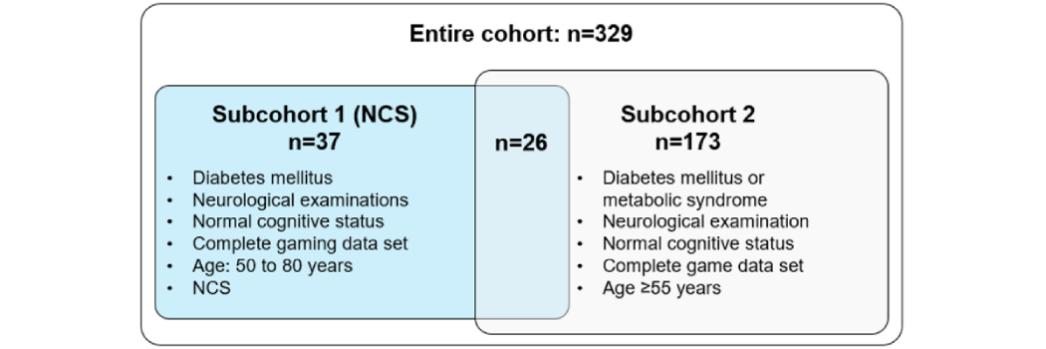
Overview of study cohorts. A total of 145 patients were excluded from the subgroup analyses for not meeting the predefined inclusion criteria outlined in the Venn diagram, e.g., cognitive dysfunction. NCS: nerve conduction study.

Overall, 329 patients were analyzed in the study (Figure 1). In subcohort 1, we recruited 37 patients aged 50 to 85 years and diagnosed with diabetes mellitus. They were of normal cognitive status (MoCA>25) who completed the video games and underwent NCS as outlined [33]. In subcohort 2, we analyzed 173 patients with normal cognitive status (MoCA>25 or MMSE>24) aged 55 to 85 years with complete clinical examination and video game data sets. The age, sex, weight, BMI, and diabetes type were matched between patients with PNP and those without (w/o) PNP in subcohort 2.

### Standardization of Test Procedure

**Figure 2:**
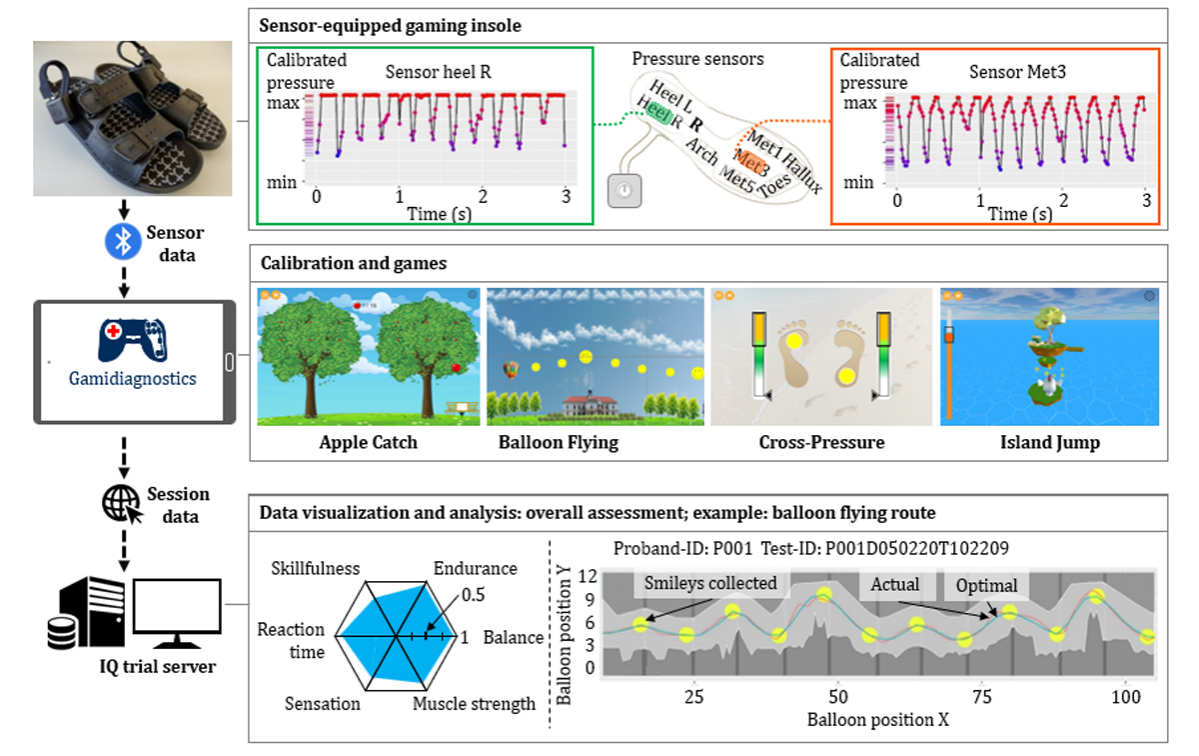
Game-based assessment of peripheral neuropathy. The sensor-equipped gaming insoles harbored 8 embedded pressure sensors in distinct areas of the plantar pedis. The insole served as a steering unit and was connected to a control unit for real-time data transmission via Bluetooth to the game application, which is run on a tablet. The setup allowed the participant to play games solely by modulating plantar pressure values. Each gaming session included 8 calibration steps and 4 games that are Balloon Flying, Apple Catch, Cross-Pressure, and Island Jump. Subsequently, the data were uploaded to the IQ trial server as a data package. The IQ trial server is a central database set up to collect and archive all data sets for further exploration. The participants received a short summary of their performance with a spider chart, scores, levels, and capabilities (beginner, average, advanced, and expert). Further data visualization for the physician and data analyses were realized to indicate the performance of participants in the different games in comparison to maximum achievement levels.

Game sessions were performed with each study participant receiving a size-matching pair of shoes harboring sensor-equipped insoles (ActiSense System, IEE S.A; Figure 2) [34]. The insoles encompassed an electronic control unit and 8 pressure sensors (5.57-cm^2^ high-dynamic HD002 force–sensing resistors) that were integrated into the heel, lateral arch, metatarsal 1, 3, and 5, hallux, and toe region of the foot (Figure S1 in Multimedia Appendix 1). The sensors allowed for pressure detection with a sensitivity of 3.4 mbar in the range of 250 mbar to 7 bar with a sampling rate up to 500 Hz, data synchronization (between insoles and smart devices), automatic detection of foot side, internal storage of 16 GB, and up to 10-hour energy supply. The sensors were embedded in foil and did not protrude. The insoles were integrated into the footwear with a support layer of ethylene-vinyl acetate-30 material and a protective layer of sponge.

**Figure 3:**
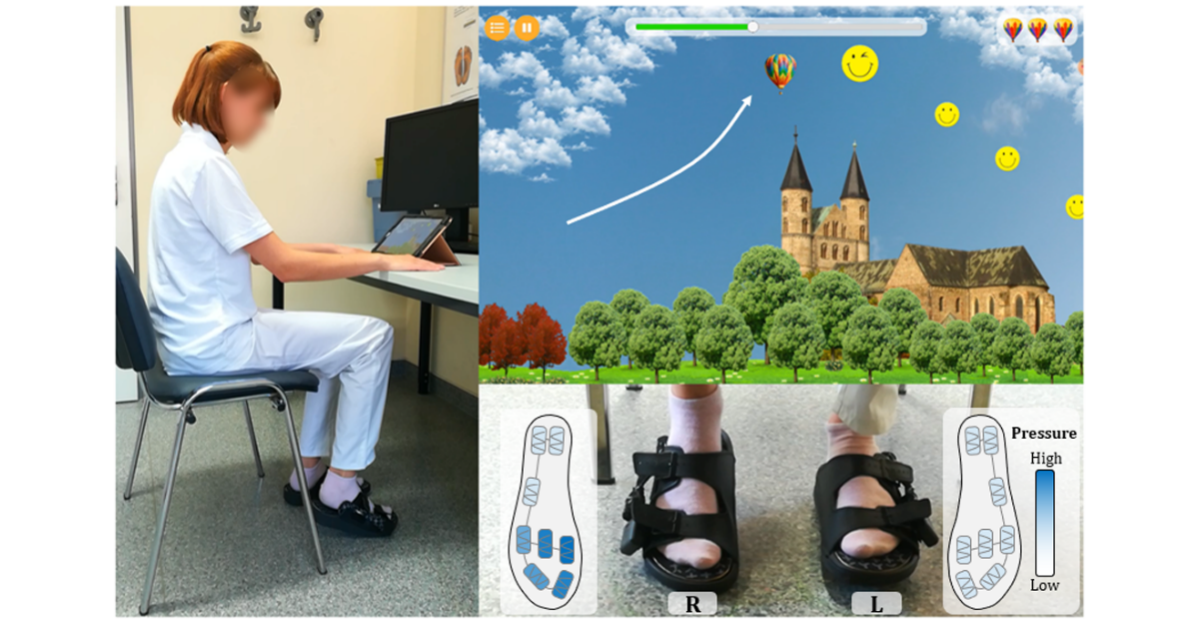
Visualization of the gaming session setup (Balloon Flying). The study participant is instructed to wear a pair of size-matching slippers with pressure sensor–equipped insoles and is seated on an armless chair with hands resting on the table (left image). The insoles serve as control units for the video-based gaming application that is run on a tablet (here the Balloon Flying game image is shown in the top right). The task is to collect as many smileys as possible by modulating the plantar pressure (bottom right image).

During the gaming session (Figure 3), sensor data were recorded at 200 Hz and transferred in real time to the application via Bluetooth (5.0) for smooth steering of games. During the test, the participants were seated on a chair without armrests in front of a table on which an Android tablet (Samsung Galaxy Tab A T580) was positioned that connected to the insoles. The participants initially calibrated the insoles through 8 standardized steps [34]. The measured pressure thresholds were used to normalize the absolute pressure values to the range of 0 to 1. Subsequently, participants familiarized themselves with the setup and control techniques of the video games through standardized tutorials.

Each gaming session consisted of 4 games: Apple Catch (AC), Balloon Flying (BF), Cross-Pressure (CP), and Island Jump (IJ), which were played sequentially (Figure 2). An introduction video for the gaming application is provided as Multimedia Appendix 2. Each session lasted about 15 minutes. The acquired insole sensor data and game outcomes were transferred in a complete data package to a remote server for data visualization and analyses. A spider chart provided feedback on the game performance to participants after the session (key capabilities: reaction time, sensation, skillfulness, muscle strength, balance, and endurance).

### Game Design and Challenges

Detailed game design and challenges have been published in the previous work [34]. Briefly, common elements of games were combined in the application, which were playful challenges in a digital setting. The steering control unit was implemented through pressure sensor–equipped insoles. The game design and challenges herein addressed the following requirements: (1) a low complexity setup allowed a quick entry into all games and an easy understanding of control functions through pressure measuring insoles. (2) Motivational elements encouraged completion of tasks and endurance over 15 minutes. (3) Standardized calibration steps and tutorials before each game allowed initial steering attempts to familiarize with the games. Tutorials were repeated on demand. (4) Standardized data acquisition processed with time stamps link sensor data over the course of games, even in the event of failed efforts (maximum allowance of 3 failed efforts per game). Comparison of data sets through time frames was maintained. (5) Definition of distinct challenges in each game provided information on movement control of both feet and legs with variables affected by muscle strength, sensation, balance, and coordination. These variables were tested for each foot separately and both feet in concert. (6) Immediate feedback to the participants on gaming results and overall performance was provided.

To normalize pressure values according to weight and maximum pressure applied by the participants, 8 calibration steps were performed before the commencement of the games [34]. These steps included the application of minimal and maximal pressure at all positions. Furthermore, the participants were instructed to stand up for 5 seconds and keep balance on each foot alone for 5 seconds. The steering unit of the insoles was programmed to apply normalized values in the games, which is minimal-maximal normalization with the transformation of all values to the range of 0 to 1.

Special care was taken in the design of the tutorials for the games. These provided standardized instructions on how to proceed with the games, allowed for some early steps in handling the insoles (examples of pressure application and guiding with insoles), motivational elements (scoring system with smileys), and the possibility to repeat instructions. The remote IQ trial server stored all sensor data together with the pseudonymized data from each participant (eg, personal data, medical history, and clinical examination results) and performed sensor data interpretation (Figure 2). Moreover, it visualized the data and provided feedback to the study participants.

In the AC game, the player was situated in an autumn harvest scenario and tried to catch as many apples as possible with a carriage that was controlled by the plantar pressure application in both forefeet (sensors Met1/3/5) [34]. The apples grew in size and fell from the tree one by one at equal time intervals. The target pressure had to be adapted for the guidance of the carriage below the apple. Additionally, the player must maintain the appropriate pressure until the apple falls into the carriage; otherwise, the carriage moves out of the ideal area. Following each task, the carriage was automatically reset to the middle line. In total, 11 distinct parameters per task were defined that are the basis for player performance assessment. The length of the AC game was standardized and encompassed a total of 14 tasks (apples). Furthermore, the tasks were combined (task combination [TC]) and assigned to different groups that were of relevance for feature extraction.

In the BF game, the player guided a balloon over a skyline (Figure 3) [34]. The flying height was adjusted by the applied pressure detected at the forefoot of the right or left insole, respectively. The balloon approached the ground if no pressure was applied. The ideal flying route was paved by 12 smileys that also suited the game’s scoring system. To collect the maximum number of smileys, the player must maneuver the balloon through the skyline and preclude collisions with obstacles, such as clouds, buildings, and trees. In the case of a collision with an obstacle and the absence of corrective measures in 5 seconds, a restart was automatically initiated. The Balloon Flying parcours consisted of 12 distinct tasks, indicating 12 tasks. For each task, distinct parameters were defined to calculate the players’ performance. These included (1) the overall number of collected smileys, (2) collision frequency, (3) minimum distance of balloon to smiley and match with optimal flight position, and (4) pressure-related parameters (normalized pressure, pressure difference, pressure gradient, and pressure-time integration). For feature extraction, 4 TCs were furthermore defined for each foot side (task combination for the left foot [TCL] or task combination for the right foot [TCR] 1-4) to assess the performance by low, intermediate, and high obstacles.

In the CP game, the players were instructed to apply pressure on different foot areas (forefoot or heel) with differing target pressure levels (low or high) [34]. Low pressure was indicated by green color, high by yellow color. The actual pressure was visualized by black arrows in a pressure bar to the left and right of the displayed feet. To achieve optimal scores, the player must readily adjust the applied pressure on the corresponding plantar foot areas and maintain the correct pressure level for at least 4.5 seconds. A smiley and checkmark confirmed the accomplishment of the task. If the insole detected no valid action within 25 seconds, the game proceeded to the next task. In total, 16 tasks corresponding to 16 combinations of foot areas and ideal pressure levels were designed in the game. Distinct parameters per task were defined to calculate the players’ performance. These parameters included the anticipation time, time outside the optimal pressure zone, relaxation time, normalized pressure, pressure difference, pressure gradient, and pressure-time integration of the left or right foot. The CP game consisted of 16 tasks with target pressure levels (low vs high) and different foot areas (left or right foot, forefoot, or heel). For feature extraction, TCs were furthermore defined for each foot side (TCL or TCR 1-6) to assess the performance by different pressure levels (low vs high) and foot areas (left or right foot, forefoot, or heel).

In the IJ game, the player steered a digital bird with jump movements from island to island in an ocean until a final destiny harboring its home was reached [34]. The player adjusted the jumping distance by modulating the plantar pressure in his forefeet. The jump direction, left or right, was adjusted by the relative pressure distribution below the right and left forefoot. To achieve the optimal score, the player must adjust according to the predefined pressure values and release the pressure at once when this has been reached. If the optimal pressure was not maintained within narrow limits, the bird jumped into the water. This initiated a game restart, and the player faced the same challenge again. There was a total of 16 islands (tasks). The players’ performance was calculated by the following parameters: attempt count, deviation from ideal pressure, anticipation time, execution time, mean pressure of execution phase, normalized pressure, pressure difference, pressure gradient, and pressure-time integration. For feature extraction, 3 TCs were furthermore defined for each foot side (TCL or TCR 1-3) to evaluate the performance by different pressure levels (low, middle, or high) and foot side (left, right, or both).

### Game Feature Extraction

Feature extraction of distinct parameters from defined tasks and TCs for the 4 games was set up (Figure S2 in Multimedia Appendix 1) [34]. Game parameters calculated from each game task were considered as primary features, such as reaction time in the first task of the AC game. Apart from that, the concept of TC was introduced, that is, a set of game tasks with similar specifications or macromeasurements were combined. For example, TCL1 covered all tasks that rely on the left foot for controlling the carriage movement to catch apples. The corresponding TCR1 consisted of all tasks that rely on the right foot. The sum, mean, and SD of primary features over game tasks of TCs were treated as secondary features. For example, in the AC game, the reaction time of TCL1 was a secondary feature that was computed from the average reaction time over tasks with the left foot. Overall, 2622 distinctive parameters reflexing players’ performance in the entirety and among similar tasks were extracted for each foot side per data set (per participant).

### Statistical Analysis

Descriptive statistics are presented in proportions and frequencies for categorical variables. For continuous variables, mean (SD) and median (IQR) are used for the description of normally and nonnormally distributed data, respectively. For comparison between groups, chi-square tests were performed on categorical variables. Either 2-tailed *t* tests or Mann-Whitney *U* tests were selected depending on data distribution. In addition, the Kruskal-Wallis *H* test or 1-way ANOVA was used for comparing multiple groups. Two-sided *P* values below .05 were considered as statistically significant, with the Holm-Bonferroni method adjusting *P* values in pairwise tests [35]. Associations between variables were determined through correlation tests and linear regression using Pearson or Spearman correlation based on data distribution. Missing NCS data were excluded during the correlation analysis. Covariates between the groups were balanced using optimal full matching, which was based on propensity scores [36]. The analysis focused on estimating the average treatment effect specifically for those who received the treatment.

### Development of Predictive Models for PNP

Binary classification models were derived to identify feet with PNP within individuals diagnosed with diabetes or MetS using game parameters extracted from acquired data sets. In the preliminary stage of feature selection, game parameters exhibiting minor discrepancies across the comprehensive data set were excluded. A correlation coefficient threshold of 0.75 was set to filter out redundant game parameters with high intercorrelations [31]. For subsequent feature selection, the data set was bifurcated into test and training data sets at a 3:7 ratio. This stratified random sampling ensured class distribution conservation. Further feature selection was performed on the training data set.

Each feature was initially assessed for its correlation with the observed class via univariate statistical techniques (eg, Wilcoxon tests or 2-tailed *t* tests). Features exhibiting interclass differences underwent further model-based variable importance evaluation supported by the R caret library [37]. Multiple models were harnessed to determine feature importance, including random forest, lasso and elastic-net regularized generalized linear models (GLMNETs), support vector machines, k-nearest neighbor, neural networks, and gradient boosting machine. Some of the methods (eg, random forest) intrinsically provide variable importance metrics. For classifiers lacking this intrinsic capability, the importance was inferred via individual receiver operating characteristic curve analyses.

Subsequently, models with different subsets of the top 20 important features (ie, variances of numbers or orders of features) were tested. The aforementioned classifiers were used during modeling. The area under the receiver operating characteristic curve (AUC-ROC) was selected as the performance metric to compare the models trained with different feature combinations. A 5-fold 10 repeats cross-validation was used in training to avoid overfitting and derive a more accurate estimate of the model performance. This statistical method repeatedly divided the training data set into 5 subsets with approximately equal size 3 times. Each subset contained the same proportion of labels as the complete data set. In total, 4 of the 5 subsets were used in the model training, while the remaining subset was used for validation. The average AUC-ROC of cross-validation was considered in the grid search of parameter combination that improves the model performance the most. Ultimately, the obtained models were further applied to the hold-out testing data set to evaluate the models’ predictive performance. Classification performance was evaluated by calculating AUC-ROC, balanced accuracy, sensitivity, and specificity, as compared with a reference standard of clinical examination.

Besides, multiple classification models were trained to predict small, large, or mixed nerve fiber damage of the feet in patients with diabetes or MetS. All data sets were used for modeling, and the 5-fold 10 repeats cross-validation was applied due to limited sample size and imbalanced class distribution. The multiclass AUC-ROC was computed to evaluate the model performance [38]. Data processing, statistical computing, and machine learning algorithms were performed using R programming language (version 4.4.1; R Foundation for Statistical Computing) and related open-source libraries as well as Zstats (version 1.0; Hangzhou Yunxiang Statistical Technology Co, Ltd).

## Results

### Characteristics of Study Participants

Overall, 299 patients with diabetes and 30 individuals with MetS were enrolled into the study and completed the game sessions. Only data sampled from the first game attempt were analyzed. Patient characteristics and clinical findings are summarized in Table 1. Moderate or severe neuropathy symptoms and deficits were observed in 201 (61.1%) and 124 (37.7%) patients, respectively. In total, 10% (n=33) of enrolled participants scored <4 in NSS and >6 in NDS, indicating the presence of an asymptomatic neuropathy (Table S1 in Multimedia Appendix 1). Clinically evident PNP was identified in 247 participants (left foot: n=236 and right foot: n=234). A total of 88 (26.7%) participants were diagnosed with asymmetric affections of PNP (Table S2 in Multimedia Appendix 1).

**Table 1:** Demographic and clinical profiles of study participants.

Cohort		Overall (N=329)	NCS^a^ subcohort 1 (n=37)	Subcohort 2 (n=173)
**Sex, n (%)**				
	Male	204 (62)	16 (43.2)	107 (61.8)
	Female	125 (38)	21 (56.8)	66 (38.2)
**Age (years), median (IQR)**		67.0 (59.0-72.0)	65.0 (61.0-70.0)	68.0 (64.0-72.0)
**Weight (kg), median (IQR)**		86.0 (77.0-98.0)	85.0 (78.0-92.0)	86.0 (78.0-96.0)
**BMI (kg/m^2^), median (IQR)**		28.7 (25.5-32.5)	28.7 (26.2-32.2)	29.1 (26.1-32.3)
**Type of diabetes, n (%)**				
	No	30 (9.1)	0 (0)	29 (16.8)
	Type 1	75 (22.8)	7 (18.9)	29 (16.8)
	Type 2	224 (68.1)	30 (81.1)	115 (66.4)
**Duration of diabetes (years), median (IQR)**		13.0 (6.0-23.5)	13.0 (8.0-25.0)	12.0 (7.0-24.0)
**Neuropathy symptom score, n (%)**				
	Normal (0-2)	106 (32.2)	9 (24.3)	57 (32.9)
	Mild (3-4)	22 (6.7)	5 (13.5)	9 (5.2)
	Moderate (5-6)	70 (21.3)	7 (18.9)	35 (20.2)
	Severe (7-10)	131 (39.8)	16 (43.2)	72 (41.6)
**Neuropathy disability score, n (%)**				
	Normal (0-2)	114 (34.7)	11 (29.7)	65 (37.6)
	Mild (3-5)	91 (27.7)	16 (43.2)	52 (30.1)
	Moderate (6-8)	103 (31.3)	9 (24.3)	44 (25.4)
	Severe (9-10)	21 (6.4)	1 (2.7)	12 (6.9)
**Clinically evident PNP^b,c^, n (%)**				
	Left foot	236 (71.7)	24 (64.9)	118 (68.2)
	Right foot	234 (71.1)	26 (70.3)	120 (69.4)
	Overall	247 (75.1)	27 (73)	126 (72.8)
	Asymmetrical PNP^d^	88 (26.7)	5 (13.5)	45 (26)

^a^NCS: nerve conduction study.

^b^PNP: peripheral neuropathy.

^c^PNP was defined as clinical findings of 2 or more neuropathic symptoms, signs, or reflex abnormalities per foot side.

^d^Asymmetric (unilateral) PNP was defined as at least 1 asymmetric finding in the clinical examination.

### Game Features Analysis in Subcohort 1

**Figure 4:**
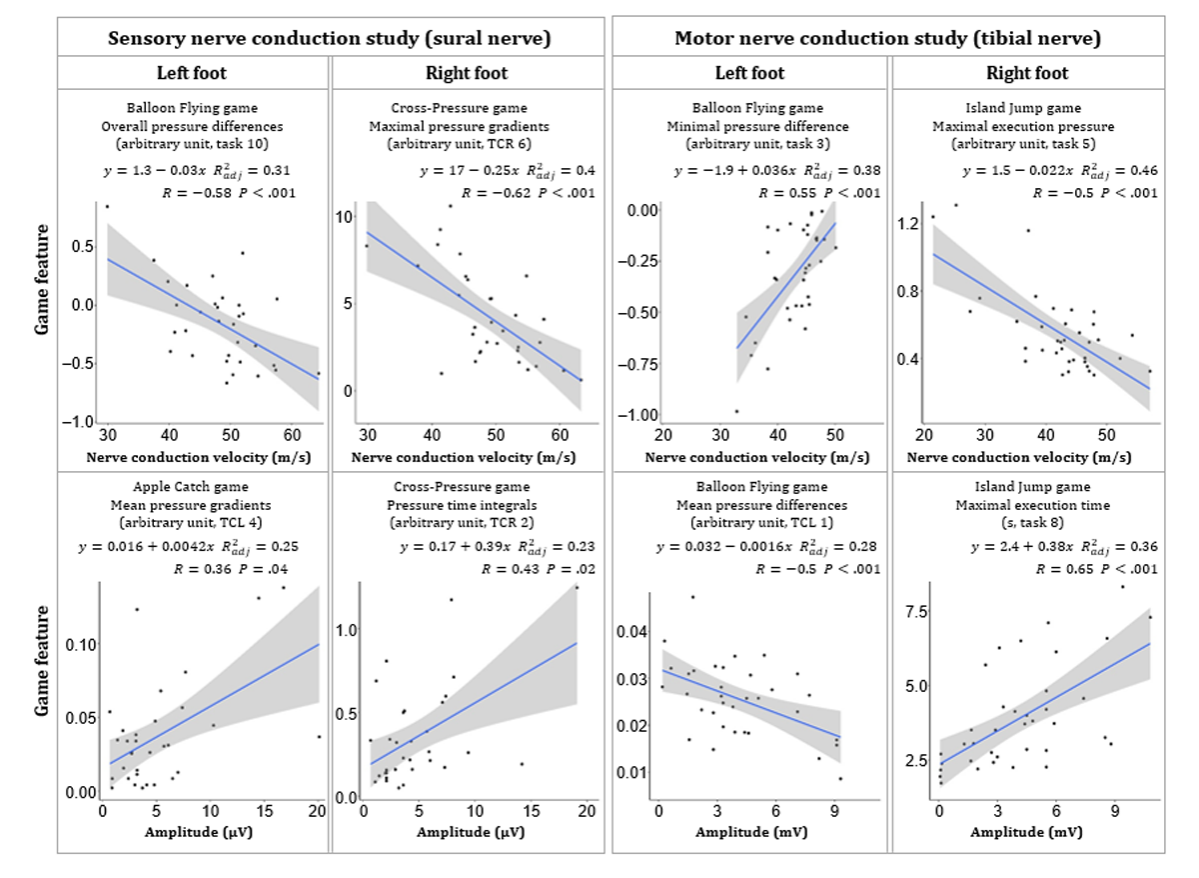
Performance in nerve conduction study and correlation with game-based findings in subcohort 1. The equation and adjusted R2 were calculated from simple linear regression models. Pearson and Spearman correlation were used for the analysis of normally and nonnormally distributed data, respectively. In total, 5 of 37 patients had incomplete nerve conduction study data. TCL: task combination for the left foot; TCR: task combination for the right foot.

NCS were performed in 37 patients (subcohort 1, aged ≥50 years, Table 1), all diagnosed with diabetes mellitus and normal cognitive status (MoCA>25). This subcohort had similar distributions in age, weight, BMI, and neuropathic scores as the entire cohort (Table S3 in Multimedia Appendix 1). The sex distribution is lightly skewed toward female patients in this subcohort. Patients’ game performance was significantly correlated to the sensory and motor NCV and amplitude in the lower extremities. Overall, 277 independent game features were significantly correlated with NCV or nerve amplitude, 79 of these presented moderate correlations (|*R*|>0.4). Eight representative features are depicted in Figure 4. The strongest correlation was observed between the motor nerve conduction amplitude of the right foot and the maximal execution time in task 8 of the IJ game (*R*=0.65; *P*<.001; adjusted *R*^2^=0.36; last plot in Figure 4).

BF and AC games contributed more features that were correlated with the NCV and amplitude of the left foot. However, more features extracted from BF and IJ games were associated with the NCV and amplitude of the right foot. In total, 164 and 113 independent game features were significantly correlated with motor and sensory nerve status, respectively. Overall, 146 independent game features were found to be associated with NCV, and 131 were correlated to the amplitude of nerves of the foot. Only 5 patients had incomplete NCS data (Table S4 in Multimedia Appendix 1).

### Game Features Analysis in Subcohort 2

Of all participants (N=329), a subcohort 2 (n=173) with normal cognitive state (MoCA>25) aged 55 years and older was generated (Table 1 and Table S5 in Multimedia Appendix 1). No significant differences in age, sex, weight, BMI, and type of diabetes were observed between patients w/o clinical PNP and with clinical PNP. Individuals with neuropathy had a significantly longer duration of diabetes and more severe neuropathic symptoms. Clinically evidence for PNP was present in 118 left and 120 right feet. For all these feet, at least 1 insensate site in the 10-g monofilament testing was detected. PNP was identified in both feet in 112 patients. No PNP was present in 47 patients, 26% (n=45) of patients had unilateral PNP.

**Figure 5:**
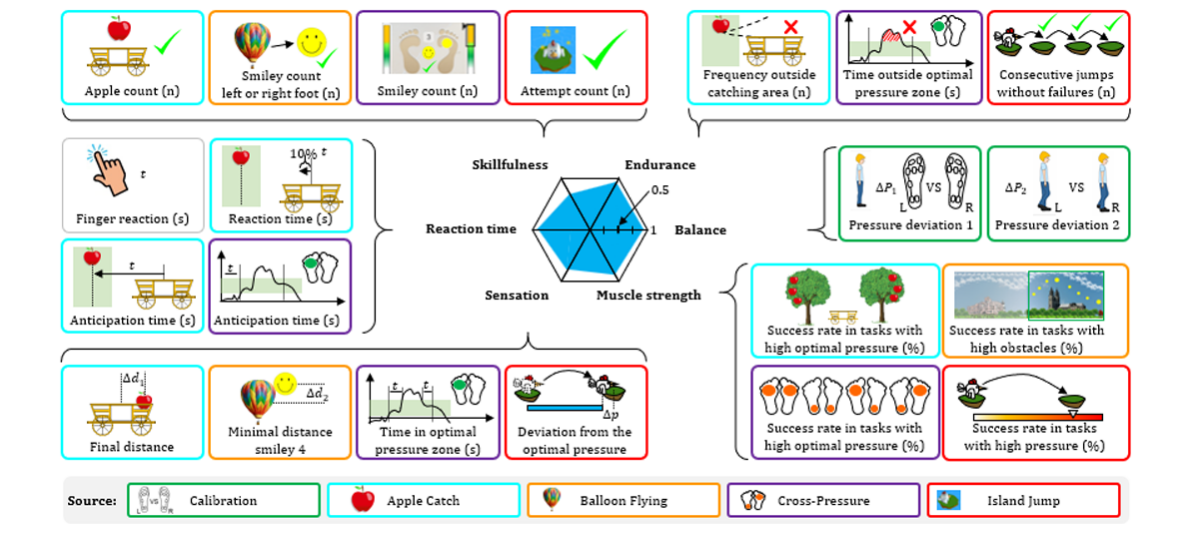
Considerations on key capabilities and scoring of video games (hypothesis-driven). Six hypothesis-driven key capabilities were identified to quantify the player’s game performance: reaction time (speed and accuracy of immediate task responses), sensation (precision in pressure application during subtasks), skillfulness (overall performance across games), muscle strength (performance in tasks requiring high-pressure application), balance (pressure distribution between left and right foot), and endurance (consistency of pressure application over time). Each key capability score is derived from various underlying game parameters and normalized to a scale from 0 to 1, with 1 representing perfect performance. The actual scores are visualized in a spider diagram.

According to the hypothesis that critical skills related to nerve functions are required for successful game performance, 6 hypothesis-driven key capabilities were defined: reaction time (understanding; immediate response to tasks), sensation (fine-tuning of pressure application in subtasks), skillfulness (overall achievements in each game), muscle strength (achievements with high-pressure application), balance (pressure distribution left vs right foot), and endurance (steadiness of pressure application in tasks). The consideration on hypothesis-driven key capabilities and scoring of video games is presented in Figure 5.

**Figure 6:**
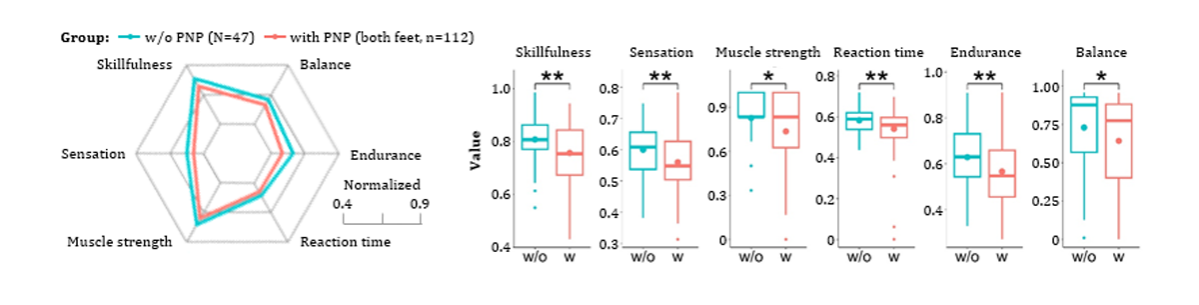
Presentation of hypothesis-driven key capabilities between participants without (w/o) and with (w) symmetrical peripheral neuropathy (PNP). Data outliers were excluded in boxplots. Significance levels: *P=.01-.05, **P=.001-.01.

**Figure 7:**
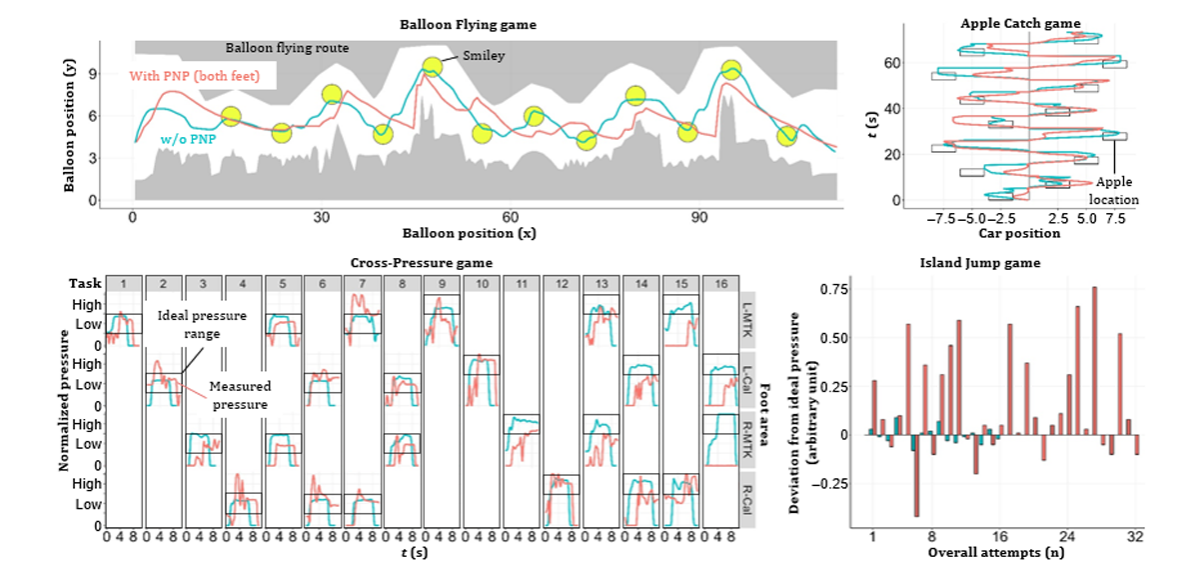
Visualization of game performance of 2 study participants (blue: healthy individual without [w/o] PNP and red: patient with symmetrical PNP). PNP: peripheral neuropathy.

**Figure 8:**
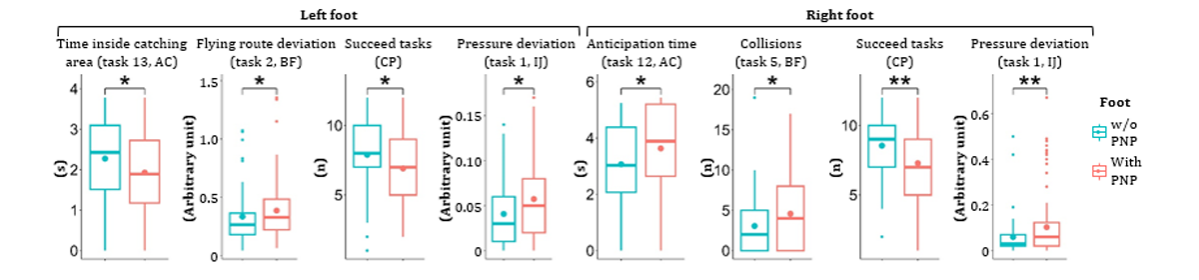
Representative game features correlated with the presence of PNP. Data outliers were excluded in boxplots. AC: Apple Catch; BF: Balloon Flying; CP: Cross-Pressure; IJ: Island Jump; PNP: peripheral neuropathy; w/o: without. Significance levels: *P=.01-.05, **P=.001-.01.

All key capabilities revealed significant differences between patients w/o PNP (n=47) and with symmetrical PNP (n=112; Figure 6). Exemplary game performance differences between 2 participants are visualized for all games (w/o PNP: blue color; with PNP: red color; Figure 7). Of 2622 extracted game features, 50 (left side) and 56 (right side) independent game features revealed significant differences between the “w/o PNP” and “with PNP” groups. Representative examples of these game features were compared and visualized for each game (Figure 8).

**Figure 9:**
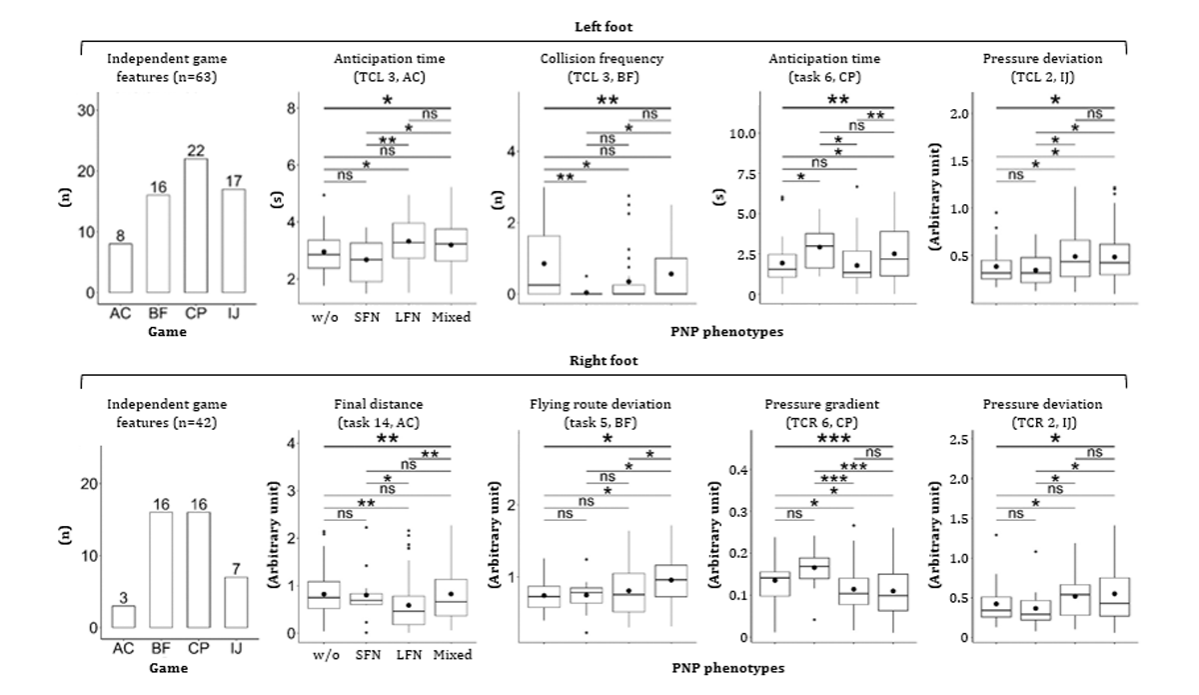
Independent game features that correlated significantly with clinical phenotypes of PNP. Data outliers were excluded in boxplots. AC: Apple Catch; BF: Balloon Flying; CP: Cross-Pressure; IJ: Island Jump; LFN: large fiber neuropathy; PNP: peripheral neuropathy; SFN: small fiber neuropathy; TCL: task combination for the left foot; TCR: task combination for the right foot. Significance levels: ns: *P*>.05, *P=.01-.05, **P=.001-.01, ***P=.0001-0.001.

### PNP Predictive Models

With the extracted game features from 173 patients in subcohort 2, predictive models for PNP were trained using the 5-fold 10 repeats cross-validation. In total, 70% (n=122) of game data sets were used for model training, and 30% (n=51) for testing. In the hold-out testing data set, the obtained GLMNET identified PNP in the left foot with an adjusted accuracy of 76.1% (sensitivity: 77.1%; specificity: 75%; and AUC-ROC: 0.72). For determining PNP in the right foot, another GLMNET model achieved an accuracy of 81.7% (sensitivity: 83.3%; specificity: 80%; and AUC-ROC: 0.75). An overview of the model’s predictive performance is presented in Tables 2 and 3.

**Table 2:** Predictive performance of peripheral neuropathy (PNP) models on the held-out testing data set (n=51).

		Left foot		Right foot
Model		GLMNET^a^		GLMNET
**Tuning parameters**				
	α	.1	.1	
	λ	0.000431594129792057	0.00209857537479028	
**Accuracy (%)**		76.1		81.7
**Sensitivity (%)**		77.1		83.3
**Specificity (%)**		75		80
**AUC-ROC^b^**		0.72		0.75

^a^GLMNET: lasso and elastic-net regularized generalized linear model.

^b^AUC-ROC: area under the receiver operating characteristic curve.

**Table 3:** Confusion matrixes of peripheral neuropathy (PNP) predictive models on the held-out testing data set (n=51).

Foot		Predicted with PNP	Predicted without PNP
**Left foot**			
	With PNP	27	8
	Without PNP	4	12
**Right foot**			
	With PNP	30	6
	Without PNP	3	12

**Figure 10:**
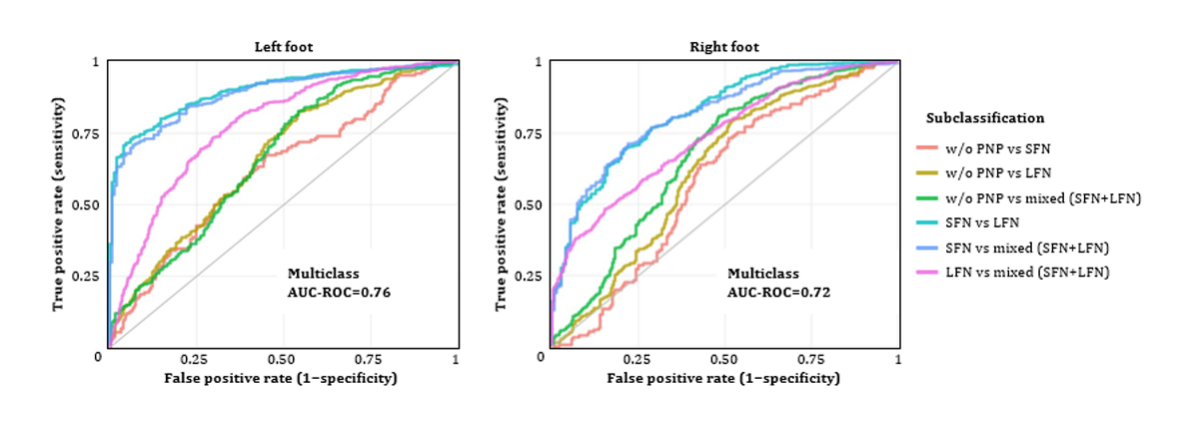
Visualization of multiclass AUC-ROC curves for the PNP subclassification (small or large or mixed nerve fiber dysfunctions). AUC-ROC: area under the receiver operating characteristic curve; LFN: large fiber neuropathy; PNP: peripheral neuropathy; SFN: small fiber neuropathy.

In addition, multiclassification models were established to differentiate feet w/o PNP versus SFN, LFN, and mixed nerve fiber damages (Table 4). Game features were extracted separately for the left and right foot from the game data set. The representative game features with significant differences between the 4 groups are depicted (Figure 9). The trained gradient boosting machine model yielded a multiclass AUC-ROC of 0.76 and 0.72 when identifying the nerve fiber damage pattern for the left and right foot, respectively (Figure 10). Both models performed better in distinguishing between SFN and LFN. Game features selected by the learning models are provided in Figures S3-S6 in Multimedia Appendix 1.

**Table 4:** Clinical phenotypes of PNPa according to clinical examinationsb.

PNP phenotypes			Without	SFN^c^		LFN^d^		Mixed
**SFN (Aδ- or C-fiber deficits, 0-2 points)**								
	Temperature sensitivity (0=normal and 1=reduced or absent)	0		1-2	0		1-2	
	Pain sensitivity (pin-prick; 0=normal and 1=reduced or absent)	0		1-2	0		1-2	
**LFN (Aα- or Aβ-fiber deficits, 0-3 points)**								
	Ankle reflexes (0=normal and 1=reduced or absent)	0		0	1-3		1-3	
	Vibration perception (128-Hz tuning fork; 0=normal and 1=reduced or absent)	0		0	1-3		1-3	
	10-g monofilament (3 test sites; 0=no insensate sites and 1=1-3 insensate sites)	0		0	1-3		1-3	

^a^PNP: peripheral neuropathy.

^b^Left feet (overall n=173): without: n=31, SFN: n=15, LFN: n=62, and mixed: n=65; and right feet (overall n=173): without: n=28, SFN: n=15, LFN: n=64, and mixed: n=66.

^c^SFN: small fiber neuropathy.

^d^LFN: large fiber neuropathy.

## Discussion

### Principal Findings

This proof-of-concept study tested the feasibility and accuracy of a game-based setup to detect PNP in patients with diabetes mellitus or MetS. The games were designed to detect key capabilities related to nerve functions with result interpretations based on hypothesis-driven assumptions as well as algorithms developed by artificial intelligence (AI) methodologies. The findings from NCS data suggested that a standardized gaming session of 15-minute length is sufficient to acquire an assessment of peripheral nerve function. The extracted game features were correlated with both NCV and amplitude of the major nerves of the foot, suggesting a possible combination of axonal degeneration and segmental demyelination in the study cohort [39]. The detected correlations of game features with NCS findings yielded maximum *R* values of 0.65. These are of similar magnitude as data sets using NDS alone (*R*=–0.69; *P*<.001) or with the application of the Toronto Clinical Neuropathy Score (*R*=–0.58; *P*<.001) [40].

Learning models trained with gaming data sets from subcohort 2 allowed for the discrimination between feet with PNP and healthy feet with accuracies of 76.1% and 81.7% for each foot side, respectively. The definition of PNP is based on the widely used diagnostic criteria (a combination of NSS, NDS, and monofilament testing) but implemented separately for each foot side [31]. Clinically evident PNP was identified in 75.1% (n=247) of study participants, suggesting a very high risk of developing foot complications in our cohort [41]. This unusual high prevalence of PNP identified in our study cohort is likely due to a patient selection bias. For the specialized outpatient clinic, referral is mostly due to inadequate blood sugar control and comorbidities with diabetes. The patients were mostly at an advanced age with a long duration of diabetes [42]. Notably, the proposed diagnostic criteria for “probable” PNP fitted into the concept of the Toronto Consensus Guidelines [32], which was applied to both feet separately following clinical examination.

In addition, the chosen diagnostic criteria according to clinical examination allowed to discriminate between nerve damage patterns of PNP. The obtained multiclassification models predicted the nerve damage pattern (SFN, LFN, or mixed fiber neuropathy) with AUC-ROC of 0.76 and 0.72 for the left and right foot, respectively. Importantly, gaming data from patients with cognitive impairment (MoCA≤25 or MMSE≤24) were excluded from analyses and AI modeling [43].

Asymptomatic neuropathy was observed in 10% (n=33) of the study participants. This underscores the importance of developing easy-to-use screening tools to avoid or delay the progression of PNP. In addition, more than one-fourth of participants were diagnosed with asymmetric neuropathy, which is rarely reported in other studies [44]. Therefore, we performed analyses and modeling separately for each foot to circumvent the methodological errors caused by asymmetric neuropathy. The results are in line with our concern and yielded different results for the left and right foot.

### Strengths and Limitations

The presented game-based approach combines several aspects inherent in screening tools recommended by the American Diabetes Association and guidelines from the Canadian Diabetes Association [5,32]. The procedure may be performed in a highly standardized manner without guidance by health care professionals, given that the tutorial is self-explanatory. The barrier to completion in this study is low, even for those with little or no gaming experience. The immediate response of the study participants upon completion of the games was overwhelmingly positive. More than 90% (n=297) wished to repeat the sessions. However, the results of the second course were not included in this study to rule out learning effects.

However, the number of patients enrolled in the study was still too low to build more refined AI models. The trained learning models using gaming data sets from the age-matched subcohort 2 could achieve binary classification for PNP with moderate accuracy. The obtained multiple classification models performed differently in identifying various clinical phenotypes of PNP. The AUC-ROC for distinguishing between SFN and LFN was obviously higher than differentiating SFN from the “w/o PNP group.” However, only a limited number of patients with pure small fiber deficits were enrolled. Small fiber deficits are believed to be precursors of large fiber impairment in PNP [19]. It remains unclear whether game-based diagnostics (ie, incorporation of game-playing elements into the diagnosis of neuropathy) will be similarly sensitive in detecting isolated Aδ- or C-fiber neuropathy. Therefore, more patients in the early stages of diabetic polyneuropathy need to be tested in subsequent studies. Notably, with early diagnosis, preventive measures may be taken, such as weight control or normalization of hyperglycemia [45].

A systematic analysis of the influences of age and cognitive impairment on the execution of the games will also be of interest. The results may set the stage for exergaming approaches and tests of learning capabilities, for example, through repetition of games [46]. A learning effect and changes in the outcome of test findings with repeated performance may not be excluded. Repeated game data sets from the same study participants (n>100) will facilitate the exploration of the influence of the learning effect and enable the testing of the robustness of the study findings.

The order of gaming tasks was also fixed in our setup, as we wished to standardize it as much as possible. Ramifications by introducing randomized sequences of gaming tasks would be of interest in follow-up studies. In addition, the handedness may confound the findings, which may not be unraveled by our analyses. The data sets for left-handedness were too scarce to bring forward a meaningful assessment. A specific cohort of left-handed individuals exceeding 50 is required to refine the findings and explore the impact of left versus right-handedness on the proposed game-based assessment. Reproducibility of findings by others will be key before generalization may be advocated.

### Future Directions

In future prospective studies, we envision further cooperation with clinicians in the fields of endocrinology and neurology to implement telemedical platforms for regular PNP assessments. Besides, given the high potential of virtual reality in medical applications, we wish to use such technologies to monitor patients’ eye movements and emotional responses in our gaming environment and explore their correlations with game performance [47].

### Conclusions

Our proof-of-concept study with a game-based tool to identify patients with PNP was successful and held the potential to ease PNP screening. The findings revealed accuracies in classifying PNP and nerve damage patterns. Playful motivational elements and automatic data interpretation may be implemented in future telemedical setups. Future testing of larger cohorts will iteratively improve the performance of the feature extraction procedure and promote AI models.

## Appendix

Multimedia Appendix 1 Subcohort analyses and visualization of game features for peripheral neuropathy classification models.

Multimedia Appendix 2 Introduction video on the game-based assessment application for peripheral neuropathy.

## Abbreviations

AC: Apple Catch
AI: artificial intelligence
AUC-ROC: area under the receiver operating characteristic curve
BF: Balloon Flying
CP: Cross-Pressure
GLMNET: lasso and elastic-net regularized generalized linear model
IJ: Island Jump
LFN: large fiber neuropathy
MetS: metabolic syndrome
MMSE: Mini-Mental State Examination
MoCA: Montreal Cognitive Assessment
NCS: nerve conduction study
NCV: nerve conduction velocity
NDS: neuropathy disability score
NSS: neuropathy symptom score
PNP: peripheral neuropathy
SFN: small fiber neuropathy
TC: task combination
TCL: task combination for the left foot
TCR: task combination for the right foot

## Data Availability

All data generated or analyzed during this study are available from the corresponding author on reasonable request.
