# Supplementary material for: Game-Based Assessment of Peripheral Neuropathy Combining Sensor-Equipped Insoles, Video Games, and AI: Proof-of-Concept Study
Source: J Med Internet Res. 2024 Oct 1;26:e52323. doi: 10.2196/52323 (PMC11480693; doi:10.2196/52323)
Supplement: Multimedia Appendix 1 [file jmir_v26i1e52323_app1.pdf]

Figure S1. Sensor-equipped insole.

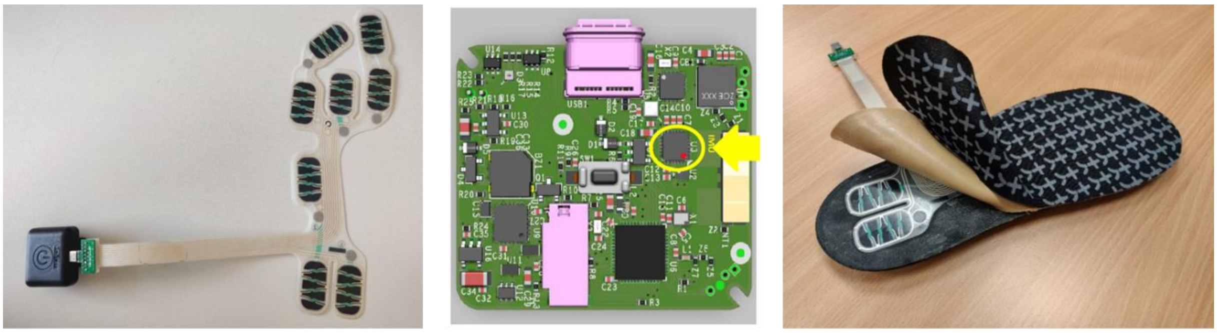

A B C

(A) The sensor-equipped gaming insole harbours eight embedded pressure sensors in distinct areas of the plantar pedis including calcaneus, arch, metatarsal (Met) 1/3/5, toes at the forefoot, and hallux at digitus 1. The insole serves as steering unit and is connected to an electronic control unit (ECU) for real-time data transmission via Bluetooth to the Gamidiagnostics application. (B) The ECU consists of nine degrees of freedom inertial measuring unit (marked with the yellow circle), Bluetooth (version 5.0), high speed processer (up to 500Hz), data synchronization (between insoles and smart devices), automatic detection of foot side, internal storage of 16 Gigabytes, and up to 10 h energy supply. (C) The insole is integrated in the footwear with a sponge protection layer and support materials of ethylene-vinylacetat-30.

Figure S2. Feature extraction from the game session data set.

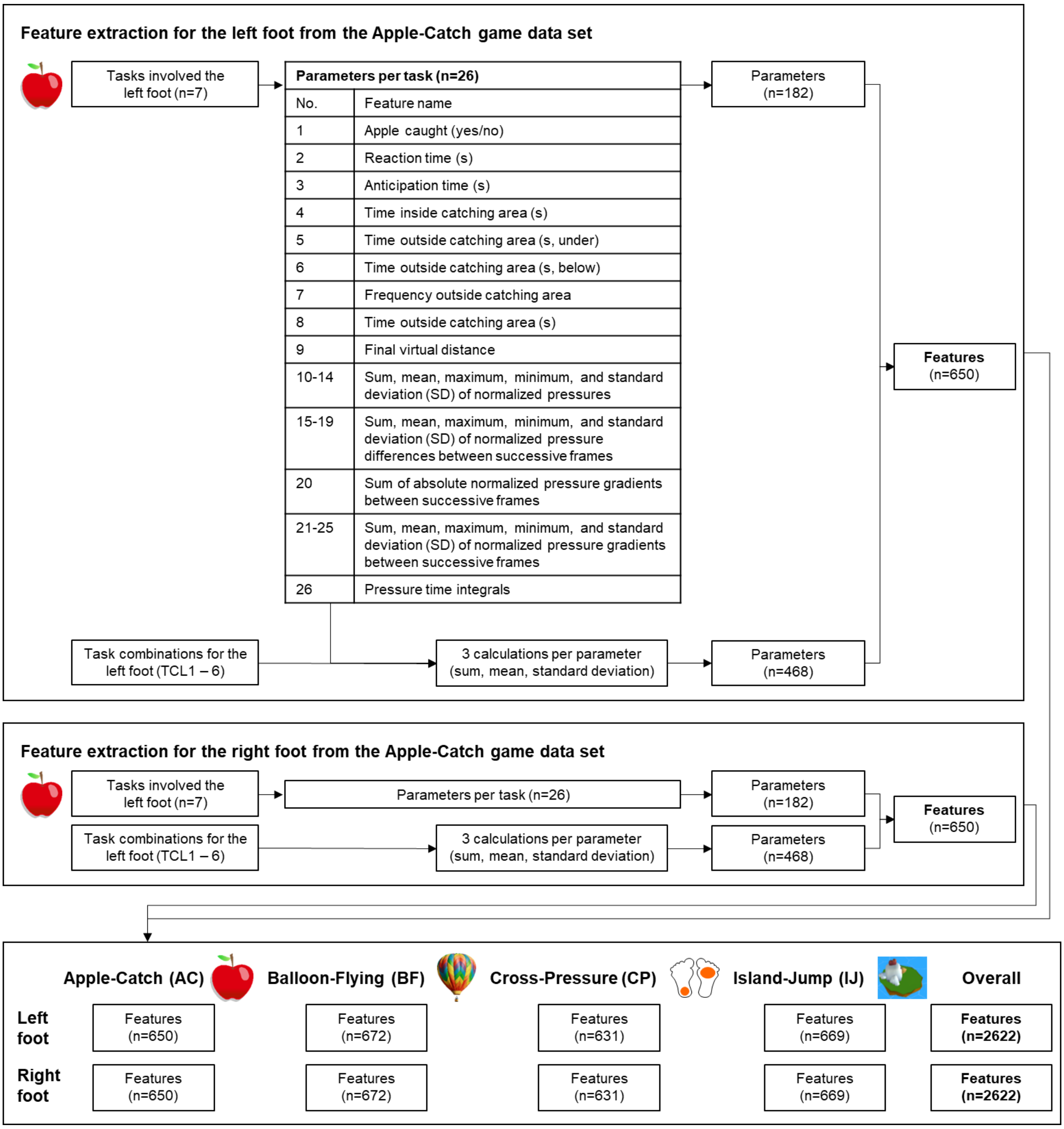

(A) Feature extraction for the left foot from the Apple-Catch game data set. Game parameters calculated from each game task were considered as primary features, such as reaction time in the first task of the AC game. The concept of task combination (TC) was introduced for each foot, i.e., a set of game tasks with similar specifications or macro-measurements were combined. (B) Feature extraction for the right foot from the Apple-Catch game data set. (C) Overall, 2,622 distinctive parameters reflexing players' performance in the entirety and among similar tasks were extracted for each foot side per data set (per subject).

Figure S3. Predictors of the PNP classification model (left Foot).

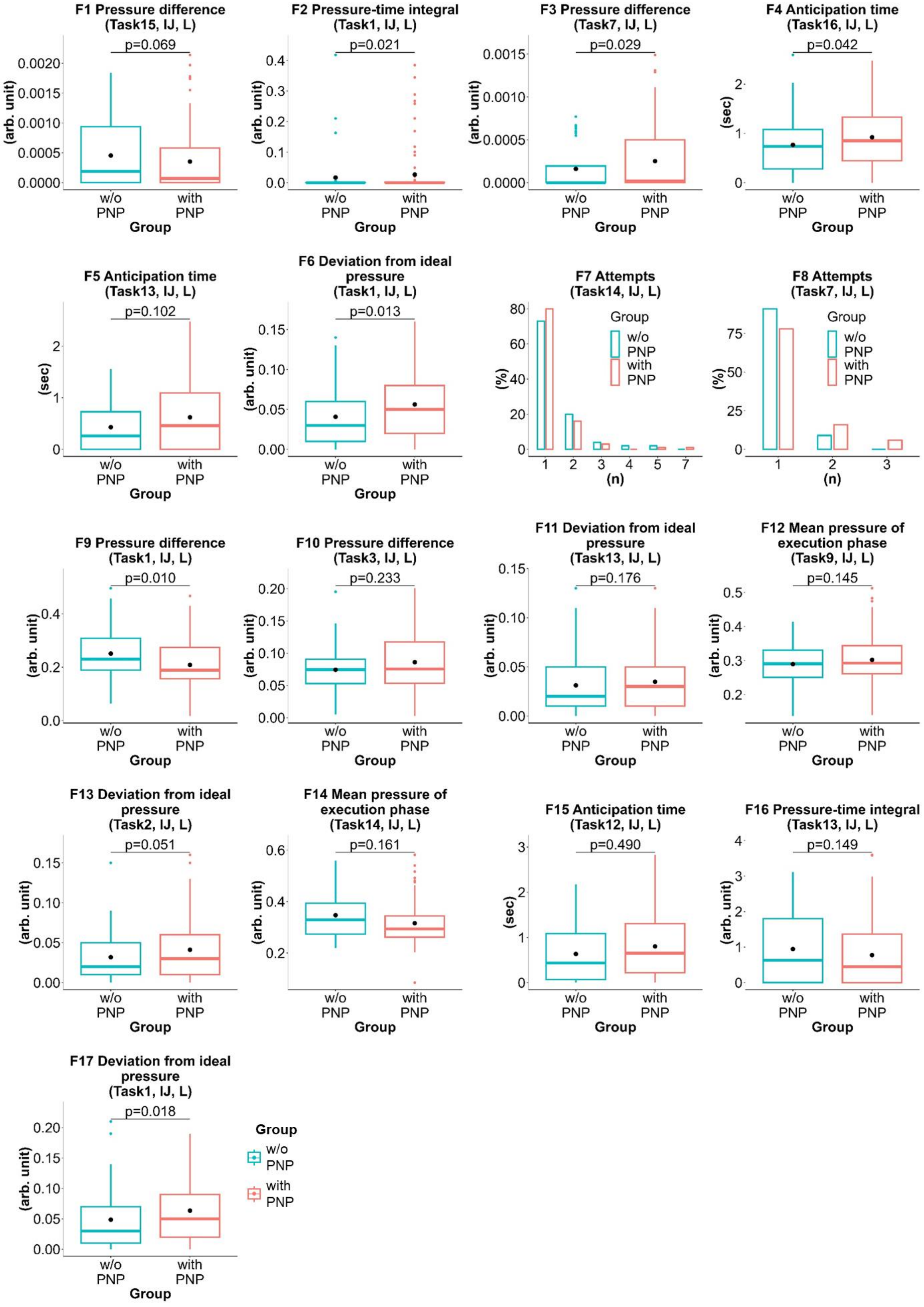

PNP: peripheral neuropathy; IJ: Island-Jump game; L: left foot; R: right foot. Data outliers were excluded in boxplots. Significance levels: ns (p>0.05), * (p=0.01-0.05), ** (p=0.001-0.01), *** (p=0.0001-0.001), **** (p<0.0001).

Figure S4. Predictors of the PNP classification model (right Foot).

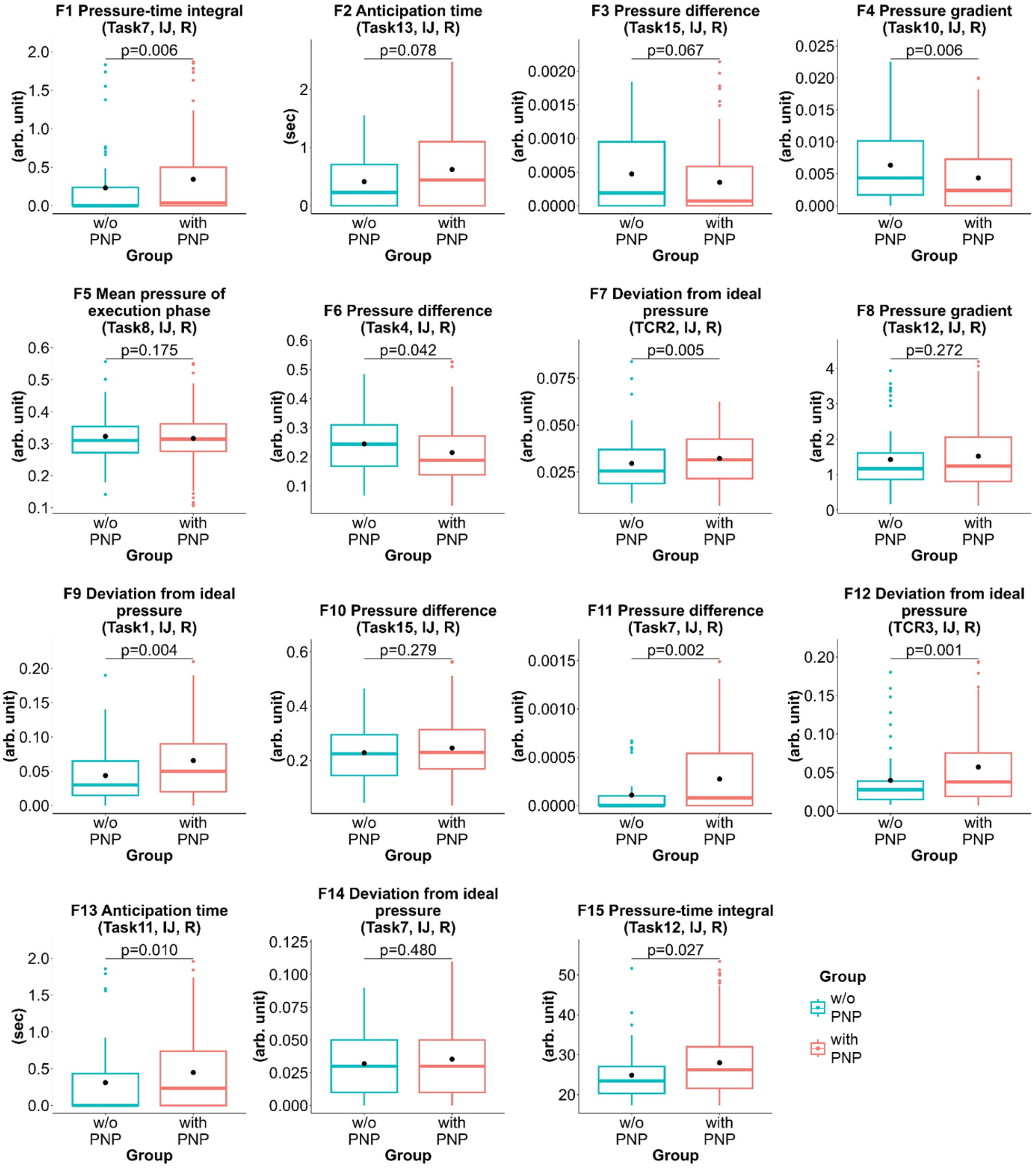

PNP: peripheral neuropathy; AC: Apple-Catch game; BF: Balloon-Flying game; CP: Cross-Pressure game; IJ: Island-Jump game; TCL: task combination for the left foot; TCR: task combination for the right foot; L: left foot; R: right foot. Data outliers were excluded in boxplots. Significance levels: ns (p>0.05), * (p=0.01-0.05), ** (p=0.001-0.01), *** (p=0.0001-0.001), **** (p<0.0001).

Figure S5. Predictors of the PNP subclassification model (left foot).

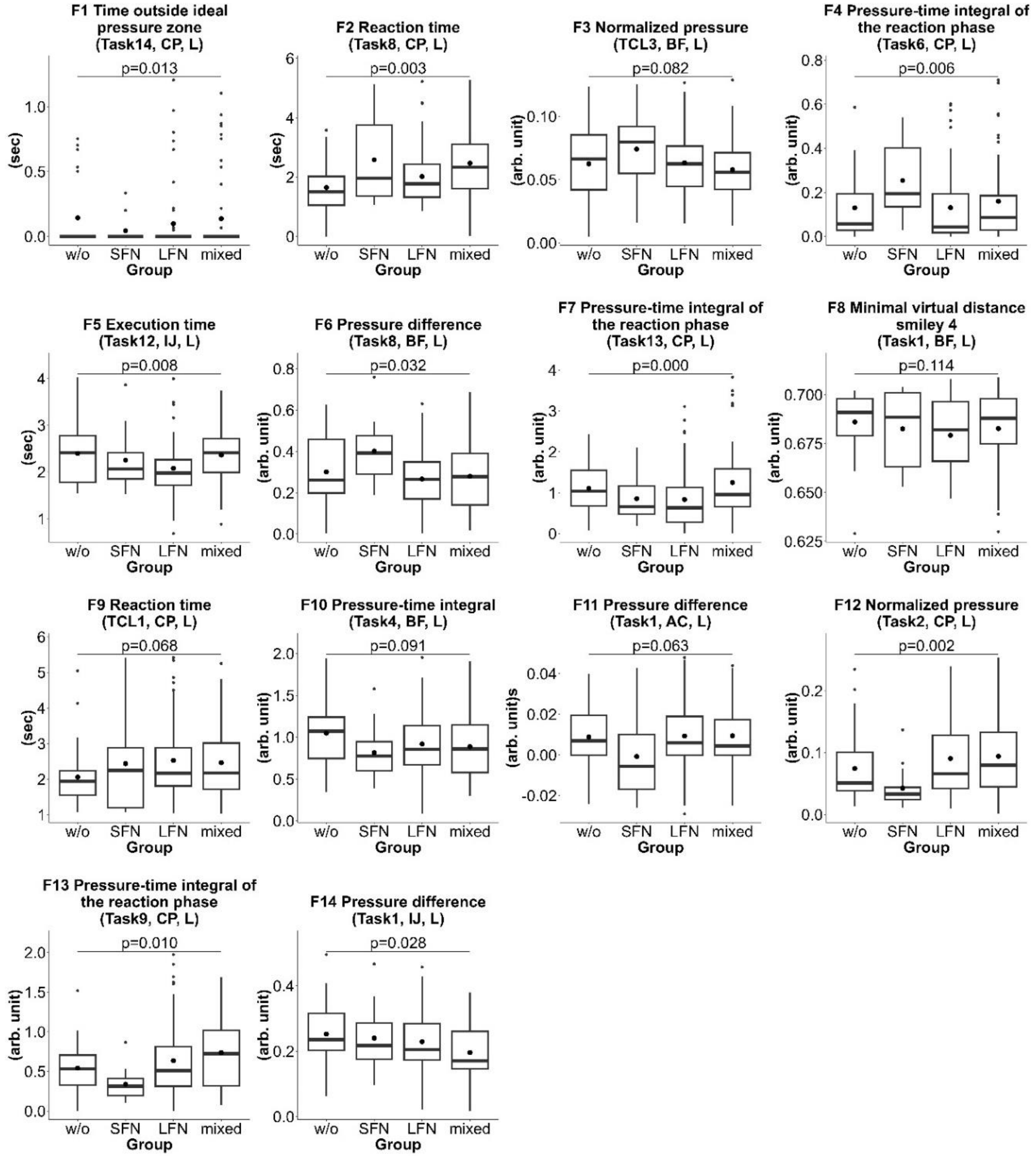

PNP: peripheral neuropathy; SFN: small fiber neuropathy; LFN: large fiber neuropathy; AC: Apple-Catch game; BF: Balloon-Flying game; CP: Cross-Pressure game; IJ: Island-Jump game; TCL: task combination for the left foot; TCR: task combination for the right foot; L: left foot; R: right foot. Data outliers were excluded in boxplots. Significance levels: ns (p>0.05), * (p=0.01-0.05), ** (p=0.001-0.01), *** (p=0.0001-0.001), **** (p<0.0001).

Figure S6. Predictors of the PNP subclassification model (right foot).

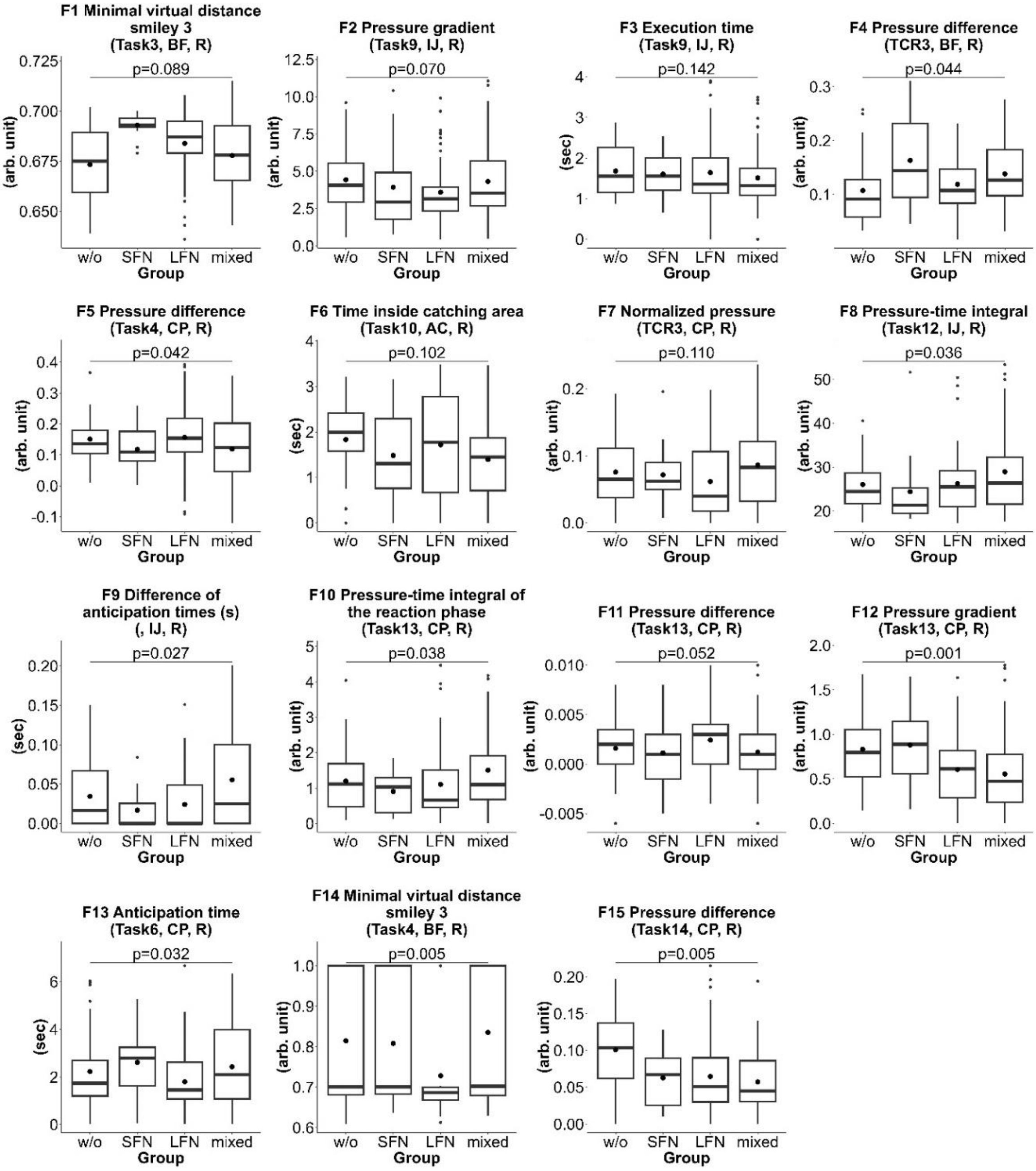

PNP: peripheral neuropathy; SFN: small fiber neuropathy; LFN: large fiber neuropathy; AC: Apple-Catch game; BF: Balloon-Flying game; CP: Cross-Pressure game; IJ: Island-Jump game; TCL: task combination for the left foot; TCR: task combination for the right foot; L: left foot; R: right foot. Data outliers were excluded in boxplots. Significance levels: ns (p>0.05), * (p=0.01-0.05), ** (p=0.001-0.01), *** (p=0.0001-0.001), **** (p<0.0001).

Table S1. Asymptomatic PNP in the entire cohort

Overall N=329		Neuropathy disability score (NDS)			
		Normal (0-2)	Mild (3-5)	Moderate (6-8)	Severe (9-10)
Neuropathy symptom score (NSS)	Normal (0-2)	55	28	20	3
	Mild (3-4)	6	6	6	4
	Moderate (5-6)	22	20	25	3
	Severe (7-10)	31	37	52	11
Asymptomatic patients (N, %)		33 (10%)			

Table S2. Clinical findings of study participants (per foot side).

Cohort	Overall (N=329)		NCS Subcohort 1 (N=37)		Subcohort 2 (N=173)	
**Foot**	Left	Right	Left	Right	Left	Right
**Positive findings (reduced/absent)**						
Temperature sensation	127 (38.6%)	136 (41.3%)	15 (40.5%)	16 (43.2%)	72 (41.6%)	76 (43.9%)
Pain sensation (pinprick)	64 (19.5%)	68 (20.7%)	10 (27.0%)	11 (29.7%)	30 (17.3%)	32 (18.5%)
Vibration perception§	219 (66.6%)	219 (66.6%)	29 (78.4%)	31 (83.8%)	114 (65.9%)	120 (69.4%)
Ankle reflex	182 (55.3%)	181 (55.0%)	16 (43.2%)	15 (40.5%)	82 (47.4%)	81 (46.8%)
10-g monofilament test	64 (19.5%)	70 (21.3%)	19 (51.4%)	18 (48.6%)	25 (14.5%)	28 (16.2%)
**Positive symptoms/signs (n)**						
0-1	93 (28.3%)	95 (28.9%)	13 (35.1%)	11 (29.7%)	55 (31.8%)	53 (30.6%)
2-6 (clinically evident PNP)	236 (71.7%)	234 (71.1%)	24 (64.9%)	26 (70.3%)	118 (68.2%)	120 (69.4%)
**Clinically evident PNP**	247 (75.1%)		27 (73.0%)		126 (72.8%)	
**Asymmetrical findings**						
Temperature sensation	47 (13.9%)		3 (8.1%)		26 (15.0%)	
Pain sensation (pinprick)	16 (4.9%)		1 (2.7%)		6 (3.5%)	
Vibration perception§	16 (4.9%)		1 (2.7%)		8 (4.6%)	
Ankle reflex	15 (4.6%)		0		11 (6.4%)	
10-g monofilament test	14 (4.3%)		1 (2.7%)		5 (2.9%)	
**Asymmetrical PNP (≥1 asymmetrical findings)**	88 (26.7%)		5 (13.5%)		45 (26.0%)	

NCS: nerve conduction studies; PNP: peripheral neuropathy; BMI: body-mass index; NSS: neuropathy symptoms score with higher scores indicating more severe neuropathic symptom; NDS: neuropathy disability score with higher scores indicating more severe neuropathic deficits. Categorical variables are presented as n (%). Mean (standard deviation [SD]) and median (interquartile range [IQR]) are used for description of normally and non-normally distributed data, respectively. §The vibration perception thresholds were adjusted according to patient age. Significance levels: ns ($p>0.05$), * ($p=0.01-0.05$), ** ($p=0.001-0.01$), *** ($p=0.0001-0.001$), **** ($p<0.0001$).

Table S3. Demographic and clinical profiles of the Subcohort 1 (NCS).

Cohort	NCS Subcohort 1 (N=37)	Entire Cohort (N=292, excluding Subcohort 1)	P
**Gender**			*
Male	16 (43.2%)	188 (64.4%)	
Female	21 (56.8%)	104 (35.6%)	
**Age (years)**	65.0 (9.0)	67.0 (13.0)	ns
**Weight (kg)**	85.0 (14.0)	87.0 (22.0)	ns
**BMI (kg/m^2)**	28.7 (6.0)	28.9 (7.4)	ns
**Type of diabetes**			ns
No	0 (0.0%)	30 (10.3%)	
Type 1	7 (18.9%)	67 (22.9%)	
Type 2	30 (81.1%)	195 (66.8%)	
**Duration of diabetes (years)§**	13.0 (17.0)	13.0 (17.0)	ns
**NSS**			ns
Normal (0-2)	9 (24.3%)	97 (33.2%)	
Mild (3-4)	5 (13.5%)	20 (6.8%)	
Moderate (5-6)	7 (18.9%)	64 (21.9%)	
Severe (7-10)	16 (43.2%)	111 (38.0%)	
**NDS**			ns
Normal (0-2)	11 (29.7%)	96 (32.9%)	
Mild (3-5)	16 (43.2%)	83 (28.4%)	
Moderate (6-8)	9 (24.3%)	95 (32.5%)	
Severe (9-10)	1 (2.7%)	18 (6.2%)	
**Overall clinically evident PNP (at least one foot)**	247 (75.1%)	27 (73.0%)	ns

NCS: nerve conduction studies; PNP: peripheral neuropathy; BMI: body-mass index; NSS: neuropathy symptoms score with higher scores indicating more severe neuropathic symptom; NDS: neuropathy disability score with higher scores indicating more severe neuropathic deficits. Categorical variables are presented as n (%). Mean (standard deviation [SD]) and median (interquartile range [IQR]) are used for description of normally and non-normally distributed data, respectively. Significance levels: ns (p>0.05), * (p=0.01-0.05), ** (p=0.001-0.01), *** (p=0.0001-0.001), **** (p<0.0001).

Table S4. Missing NCS data in Subcohort 1.

Patient ID	Sensory nerve conduction velocity & amplitude		Motor nerve conduction velocity & amplitude	
	Left foot	Right foot	Left foot	Right foot
**P231**	missing	missing	missing	available
**P310**	missing	missing	missing	available
**P414**	missing	missing	missing	available
**P505**	missing	avaiable	avaiable	available
**P073**	missing	missing	available	available

Table S5. Demographic and clinical profiles of the Subcohort 2.

Cohort	Subcohort 2 (N=173)					
	Left Feet			Right Feet		
	w/o PNP (n=55)	with PNP (n=118)	P	w/o PNP (n=53)	with PNP (n=120)	P
**Gender**			ns			ns
Male	29 (52.7%)	78 (66.1%)		29 (54.7%)	78 (65.0%)	
Female	26 (47.3%)	40 (33.9%)		24 (45.3%)	42 (35.0%)	
**Age (years)**	68.0 (7.5)	68.0 (7.8)	ns	68.0 (7.0)	68.0 (8.0)	ns
**Weight (kg)**	84.0 (20.0)	88.0 (16.8)	ns	83.0 (17.0)	88.0 (17.8)	ns
**BMI (kg/m^2)**	28.7 (5.9)	29.3 (6.3)	ns	28.3 (5.8)	29.4 (6.4)	ns
**Type of diabetes**			ns			ns
No	13 (23.6%)	16 (13.6%)		11 (20.7%)	18 (15.0%)	
Type 1	9 (16.4%)	20 (16.9%)		9 (17.0%)	20 (16.7%)	
Type 2	33 (60.0%)	82 (69.5%)		33 (62.3%)	82 (68.3%)	
**Duration of diabetes (years)**	10.0 (10.0)	14.0 (18.5)	*	10.0 (11.0)	14.0 (18.2)	*
**NSS**			****			****
Normal (0-2)	30 (54.5%)	27 (22.9%)		31 (58.5%)	26 (21.7%)	
Mild (3-4)	4 (7.3%)	5 (4.2%)		3 (5.7%)	6 (5.0%)	
Moderate (5-6)	13 (23.6%)	22 (18.6%)		13 (24.5%)	22 (18.3%)	
Severe (7-10)	8 (14.5%)	64 (54.2%)		6 (11.3%)	66 (55.0%)	
**NDS**			****			****
Normal (0-2)	51 (92.7%)	14 (11.9%)		48 (90.6%)	17 (14.2%)	
Mild (3-5)	4 (7.3%)	48 (40.7%)		5 (9.4%)	47 (39.2%)	
Moderate (6-8)	0	44 (37.3%)		0	44 (36.7%)	
Severe (9-10)	0	12 (10.2%)		0	12 (10.0%)	
**Positive clinical findings (reduced/absent)**						
Temperature sensation	10 (18.2%)	62 (52.5%)	****	10 (18.9%)	66 (55.0%)	*
Pain sensation (pinprick)	1 (1.8%)	29 (24.6%)	****	1 (1.9%)	31 (25.8%)	****
Vibration perception§	11 (20.0%)	103 (87.3%)	****	11 (20.8%)	109 (90.8%)	****
Ankle reflex	2 (3.6%)	80 (67.8%)	****	3 (5.7%)	78 (65.0%)	****
10-g monofilament test	0	25 (21.2%)	****	0	28 (23.3%)	***
**Positive clinical symptoms/signs**						
0-1	55 (100%)	0		53 (100%)	0	
2-6 (clinically evident PNP)	0	118 (100%)		0	120 (100%)	

NCS: nerve conduction studies; PNP: peripheral neuropathy; BMI: body-mass index; NSS: neuropathy symptoms score with higher scores indicating more severe neuropathic symptom; NDS: neuropathy disability score with higher scores indicating more severe neuropathic deficits. Categorical variables are presented as n (%). Mean (standard deviation [SD]) and median (interquartile range [IQR]) are used for description of normally and non-normally distributed data, respectively. §The vibration perception thresholds were adjusted according to patient age. Significance levels: ns (p>0.05), * (p=0.01-0.05), ** (p=0.001-0.01) , *** (p=0.0001-0.001), **** (p<0.0001).
